# Supplementary material for: Muramyl Dipeptide Administration Delays Alzheimer’s Disease Physiopathology via NOD2 Receptors
Source: Cells. 2022 Jul 19;11(14):2241. doi: 10.3390/cells11142241 (PMC9321587; doi:10.3390/cells11142241)
Supplement: Supplementary file 1 [file cells-11-02241-s001.zip › cells-1778469-supplementary.pdf]

cells MDPI

Supplementary Materials

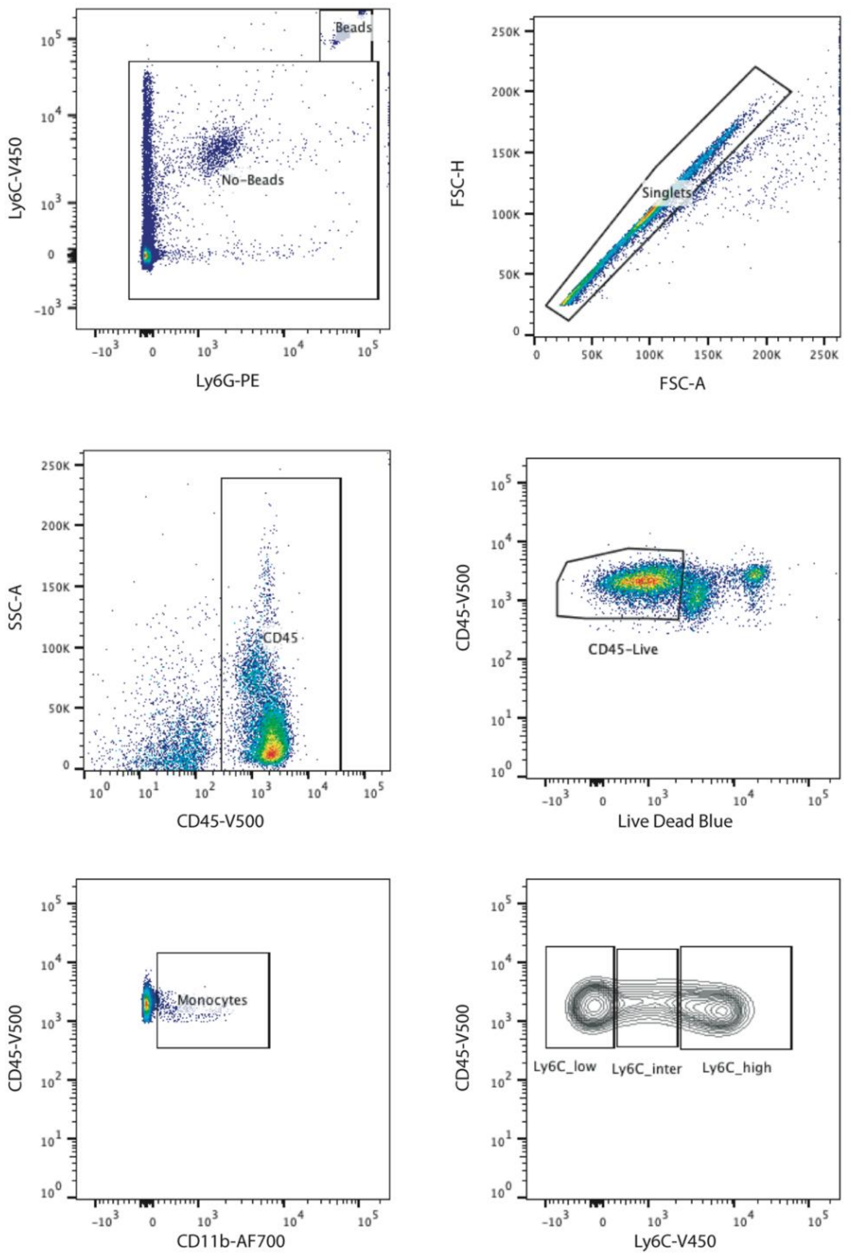

Figure S1. Flow cytometry gating strategy. Representative gating strategy for CD11b⁺ and Ly6C monocyte subsets from WT mice as measured by flow cytometry. First, 123count eBeads™ are gated with Ly6G-PE and Ly6C-V450 to determine absolute counting of cell population. After excluding the bead population, doublet discrimination is performed with a singlet gate (FSC-H/FSC-A dot blot). Only viable cells are evaluated, identified by CD45+ and low Live/dead (Blue-fluorescent reactive Dye). Next, monocytes are identified as CD45+/CD11b+. After the monocyte cell population selection, monocytes subsets were subdivided in three populations based on the Ly6C expression: $Ly6C^{hi}$, $Ly6C^{int}$ and $Ly6C^{low}$ correspond respectively to inflammatory, intermediate, and patrolling monocytes.

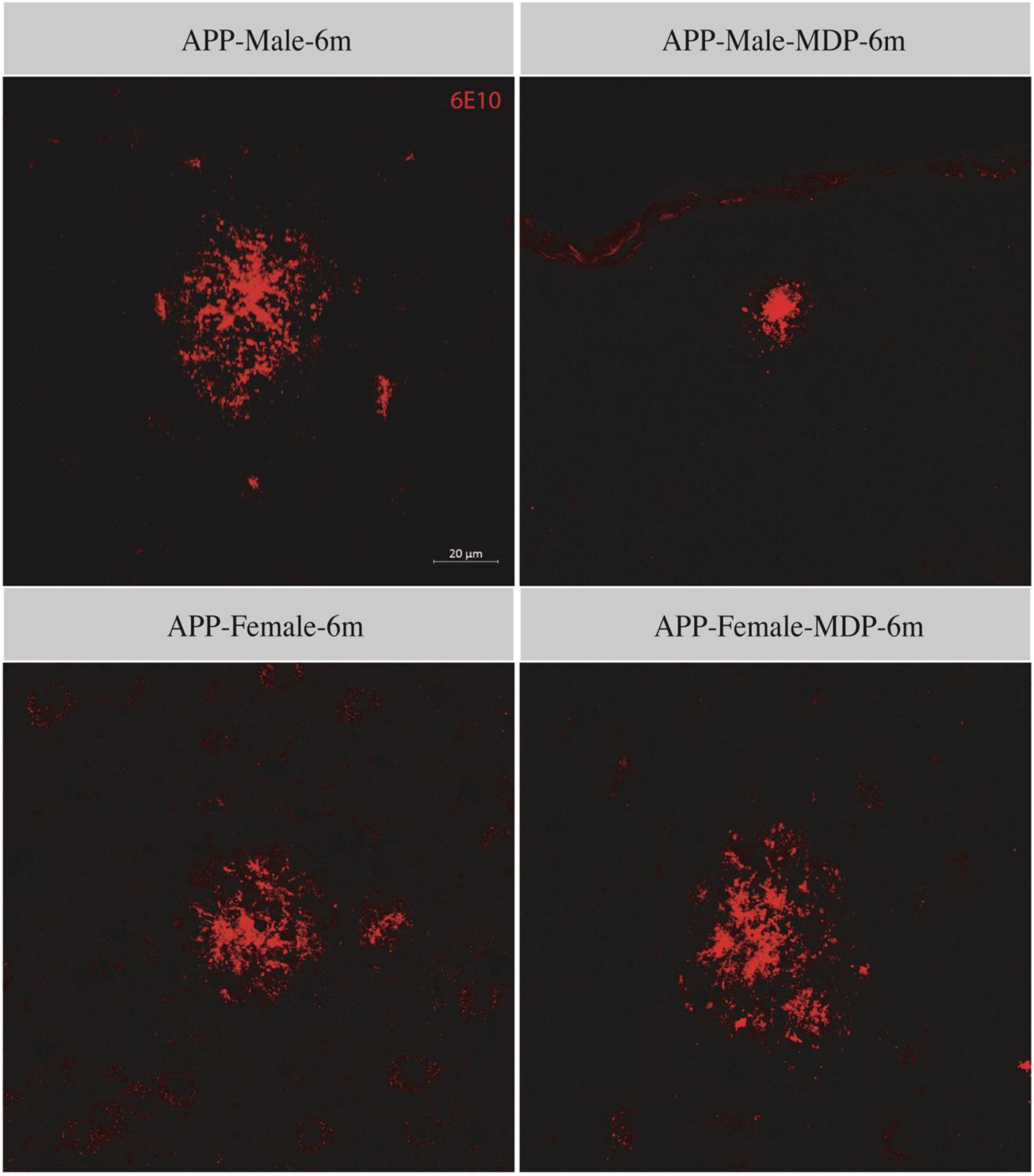

Figure S2. Representative images of 6E10 (Aβ in red) in male or female Wild-type and APP_{Swe}/PS1 treated or not with MDP from 3 to 6 month old. Scale bar = 20 µm.

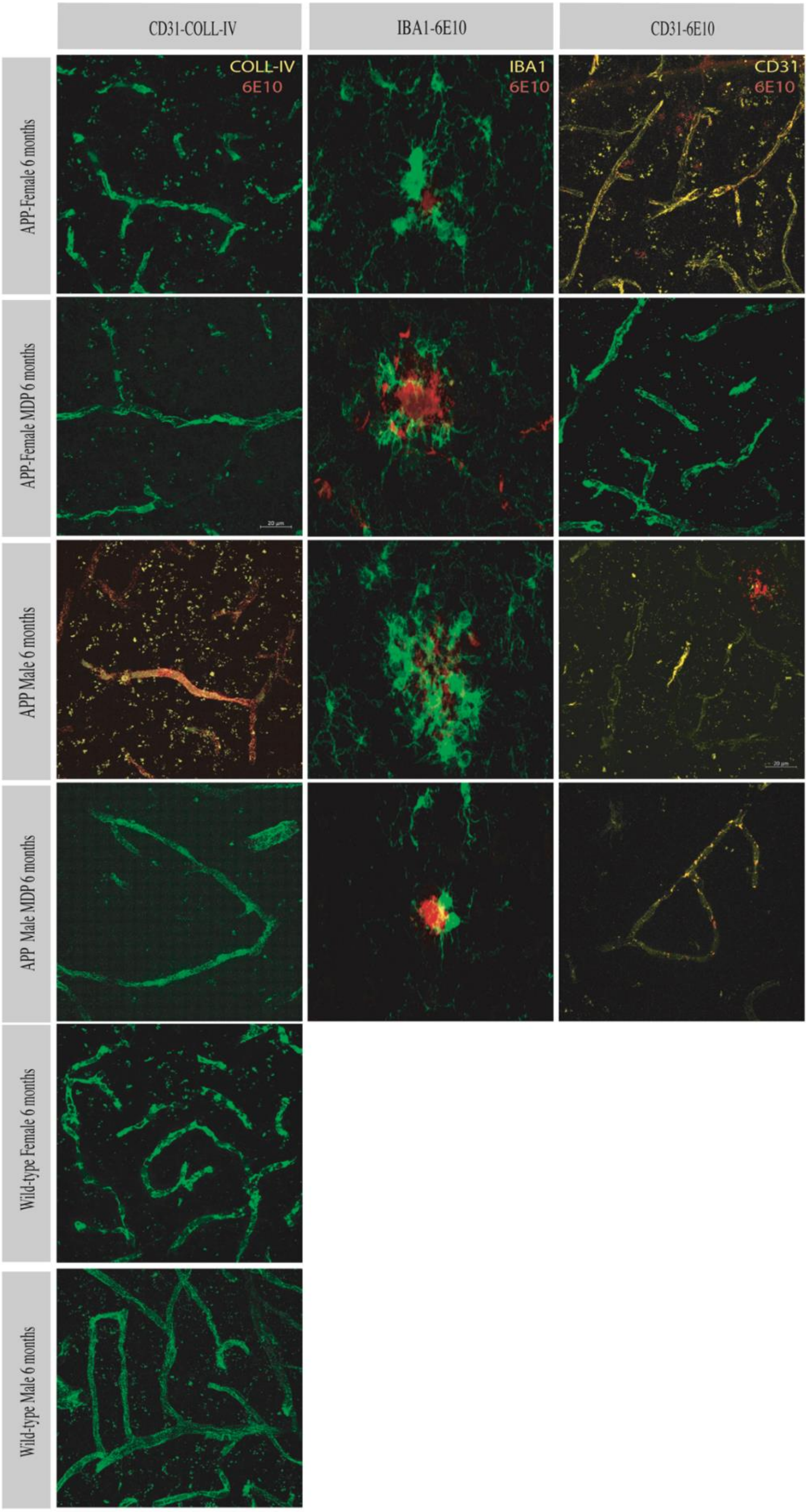

Figure S3. Representative images of CD31 (blood vessels in green) and COLLIV (red) in male or female Wild-type and APP_{Swe}/PS1 treated or not with MDP from 3 to 6 month old. Scale bar = 20 µm. Representative images of IBA-1 (microglia in green) and 6E10 (red) in male or female APP_{Swe}/PS1 mice treated or not with MDP. Representative images of CD31 (blood vessels in green) and 6E10 (red) on male or female APP_{Swe}/PS1 mice treated or not with MDP. Scale bar = 20 µm.
